# Exogenously applied spermidine confers protection against cinnamic acid-mediated oxidative stress in *Pisum sativum*

**DOI:** 10.1016/j.sjbs.2021.02.052

**Published:** 2021-02-20

**Authors:** Riti Thapar Kapoor, Mohammed Nasser Alyemeni, Parvaiz Ahmad

**Affiliations:** a Plant Physiology Laboratory, Amity Institute of Biotechnology, Amity University, Noida 201 313, Uttar Pradesh, India; b Botany and Microbiology Department, College of Science, King Saud University, Riyadh, Saudi Arabia

**Keywords:** Antioxidants, Cinnamic acid, Growth, *Pisum sativum*, Spermidine, BSA, Bovine serum albumin, CA, cinnamic acid, CAT, catalase, EC, electrolyte leakage, EDTA, ethylene diamine tetra acetic acid, GPX, guaiacol peroxidase, IAA, indole-3-acetic acid, NBT, nitro blue tetrazolium, N-1-NEDD, n-1-naphthyl-ethylene diamine dihydrochloride, NR, nitrate reductase, PA, polyamine, ROS, reactive oxygen species, RWS, relative water content, SOD, superoxide dismutase, SPD, spermidine

## Abstract

This study investigated the stress responses of cinnamic acid (CA) in pea plants and explored the protective role of spermidine (SPD) against CA-induced adverse effects. Pea seedlings exposed to CA had reduced length, biomass, moisture, chlorophyll, sugar, and protein contents and reduced nitrate reductase activity. These parameters increased when SPD was applied alone and in combination with CA. Electrolyte leakage and malondialdehyde content were high in seedlings treated with CA but decreased when the SPD + CA treatment was applied. Foliar exposure to SPD partially mitigated CA-induced stress effects by strengthening the antioxidant defense system, which helped preserve the integrity of biochemical processes. These results indicate that SPD (1 mM) could mitigate the adverse effects of CA and enhance plant defense system. Hence, SPD can be used as a growth regulator for the maintenance of physiological functions in pea plants in response to the pernicious consequences of CA stress.

## Highlights

• Cinnamic acid exposure reduced growth, pigment and protein contents but spermidine exposure attenuated the reduction.
• The accumulation of osmolytes such as sugar and proline buttressed free radicals scavenging process.
• Antioxidant metabolism was upregulated by the treatment of spermidine, thereby ameliorating cinnamic acid-induced oxidative damage.

## 1 Introduction

Plants survive in their natural habitat under a variety of environmental constraints that reduce growth and productivity. Allelopathy is a biochemical interaction between plants in which the allelochemicals released by a donor are beneficial or detrimental for the growth of its neighbors. Allelopathy was initially thought to occur exclusively between plants, but recent research revealed that allelochemicals affect all living organisms surrounding the recipient plants (Schandry and Becker, 2020). Allelochemicals are released from roots, stems, and leaves or decomposing plant residues and have phytotoxic effects on other crops. They can disrupt biochemical phenomena, including water and mineral absorption, water utilization, and pigment, protein, and ATP syntheses (Weir et al., 2004). Cinnamic acid (CA) is a phenolic acid used as an allelochemical model (Yadav et al., 2018); it alters the overall developmental and physiological regulation mechanisms of plant (Cruz-Ortega et al., 2002, Hussain and Reigosa, 2012). Ng et al. (2003) observed that the presence of CA significantly reduced the length and biomass of soybean seedlings. Zhang et al. (2010) reported that CA had adverse effects on the germination, growth, and antioxidant activity of tomato seedlings, which lead to lipid peroxidation. CA also limited the growth of *Vicia faba* by reducing the number of leaves per plant, plant height, and root and shoot weight (Zhao et al., 2018).

Polyamines are low-molecular-weight, aliphatic, and nitrogen-containing compounds (Chen et al., 2019). They have essential functions in plant growth, physiological processes, cell membrane stability, nucleic acid synthesis, and stress tolerance (Mustafavi et al., 2018, Ali et al., 2020). Free-state polyamines combine with phenolic compounds to form binding polyamines that act as secondary metabolites, which promote growth and protect plants against stress-induced changes (De Oliveira et al., 2018, Alcázar et al., 2020). Free polyamines are positively charged and can bind acidic molecules, including proteins, phospholipids, DNA, and RNA. Therefore, they influence crucial developmental processes, including cell division, DNA replication, gene transcription, enzyme activity, and membrane stability (Igarashi and Kashiwagi, 2015). Triamine spermidine (SPD) is a small, ubiquitous, naturally occurring polycationic polyamine that acts as a growth regulator involved in biochemical processes (Roychoudhury et al., 2011). It is produced and accumulated in different plant organs and plays significant roles in the defense against the toxicity caused by heavy metals, water and salt stresses, high and low temperatures, and UV radiation (Cheng et al., 2012, Upadhyay et al., 2020). SPD also inhibits the production of free radicals in plants under environmental stress (Sobieszczuk-Nowicka, 2017, Alcázar et al., 2020).

The pea (*Pisum sativum* L.; Family: Fabaceae), also named green pea, is an important vegetable in human diets owing to its sugar, protein, fiber, vitamin, and mineral contents. In addition, it has many medicinal applications due to antibacterial, antifungal, antimicrobial, antioxidant, and antitumor properties (El-Feky et al., 2018). Exposure to CA causes extensive biochemical changes in pea seedlings. Various approaches have been tested to increase plant tolerance to environmental stressors, including allelochemicals. Polyamine treatments are environmentally safe methods for efficiently attenuating the effects of toxic allelochemicals. However, previous work did not investigate if SPD supplementation can protect pea plants from the adverse effects of CA. Here, we evaluated the impact of exogenously applied SPD on pea seedlings treated with CA by analyzing growth parameters, biochemical components, and antioxidant enzyme activities.

## 2 Materials and methods

### 2.1 Pea seeds and chemicals

Healthy pea seeds (*Pisum sativum* cv. Pusa Shree) were obtained from IARI, New Delhi. The seeds were kept in airtight plastic containers to check for microbial infections. CA (molecular weight:148.161 g∙mol^−1^) was purchased from LOBAChemie Pvt. Ltd., and Spermidine (SPD; molecular weight:145.25 g∙mol^−1^) from Merck.

### 2.2 Experimental plan

Pea seeds were decontaminated by washing with 0.01% HgCl_2_ for 7 min and then cleaned with autoclaved double-distilled water and soaked for 5 h. Seeds were sown in plastic pots (12 cm height × 7 cm diameter) containing decontaminated soil moistened with a half-strength Hoagland solution. Seeds were germinated in growth chambers at 25±2°C, with 90% humidity, 16:8h light/dark cycles, and 150 μmol photons m^−2^ s^−1^; pots were irrigated with Hoagland solution on every second day. After 1 week, the number of seedlings was lessened to four per pot. Lethal doses of CA (1mM, CA1; 1.5 mM, CA2) were used to investigate the capacity of SPD to protect from CA damage.

Pea plants were treated with CA alone and in combination with SPD. The seedlings were treated 20 days after sowing. SPD was applied to foliage after mixing with Tween-20, 1 h after CA application. The seedlings were harvested 6 days after treatments, cleaned with tap water after uprooting from pots, and used to analyze the morphological and biochemical variables.

### 2.3 Determination of growth variables and relative water content

Growth assessment was performed by measuring the seedling length and biomass. The leaves were pierced into same-sized samples, and the fresh biomass was measured by weighing (FW). Subsequently, samples were kept on distilled water at 23±1°C  under dark conditions, and the turgid weight was determined after 12 h (TW). For dry biomass measurements, the leaves were kept in an oven at 60 °C for 48 h prior to weighing (DW). Relative water content (RWC) was determined using the formula RWC (%) = [(FW − DW)/(TW − DW)] * 100, in which FW is the fresh weight, TW is the turgid weight, and DW is the dry weight.

### 2.4 Pigment content

Leaf material (100 mg) was ground in acetone, and pigments (chlorophyll and carotenoid) were separated and centrifuged. The absorbance of the supernatant was recorded at 663, 646, and 470 nm, and chlorophyll *a* and b, total chlorophyll, and carotenoid contents were calculated using the method described by Lichtenthaler (1987).

### 2.5 Nitrate reductase activity and sugar, protein, and proline contents

Soluble sugars were measured using the method described by Hedge and Hofreiter (1962). Pea leaves (100 mg) were grounded in 95% ethanol. Supernatant (1 mL) was mixed with anthrone reagent (4 mL) and heated for 15 min. Optical density was measured at 620 nm, and sugar content was determined using a glucose standard curve.

To determine the protein content, 10 mg of leaf tissue was crushed in sodium hydroxide (1 N) at 100 °C for 5 min. After the addition of the alkaline copper reagent, samples were placed at room temperature for 15 min. Absorbance was measured at 650 nm, 30 min after the addition of the Folin–Ciocalteu reagent. The protein content was measured with a BSA standard curve (Lowry et al., 1951).

Nitrate reductase enzyme activity was analyzed using a modified version of the method described by Jaworski (1971). Leaf tissue (250 mg) was kept in a medium containing phosphate buffer (100 mM), 3% KNO_3_, and 5% propanol. The sample was mixed with 3% sulfanilamide in 3 N HCl and 0.02% N-1-NEDD. Optical density was calculated at 540 nm, and the nitrate reductase activity was determined using a sodium nitrite standard curve and expressed as μmol NO_2_ g^−1^ FWh^−1^.

Proline content was determined using the method previously described by Bates et al. (1973). Leaf samples were treated with sulfosalicylic acid, and acid ninhydrin and acetic acid were added to the supernatant and heated for 60 min at 100 °C. Toluene was added, and the optical density was recorded at 520 nm.

### 2.6 Determination of electrolyte leakage, hydrogen peroxide content, and lipid peroxidation

The method described by Lutts et al. (1996) was employed for electrolyte leakage analysis in pea leaves. Leaf tissue (100 mg) was incubated with distilled water (15 mL) for 24 h at room temperature. Initial electrical conductivity (EC1) was calculated when the samples were heated for 20 min. EC2 was recorded after complete cooling at room temperature. Electrolyte leakage was determined as EC = EC1/EC2 × 100.

The procedure described by Velikova et al. (2000) was employed to assess hydrogen peroxide (H_2_O_2_) content. Optical density was recorded at 390 nm, and H_2_O_2_ content was calculated from a standard curve.

Lipid degradation was analyzed by assessing malondialdehyde (MDA) content in leaves (200 mg). The leaves were grounded in 0.1% trichloroacetic acid and centrifuged at 10,000*g* for 10 min. Supernatant (1 mL) was added to 0.5% TBA and heated at 95 °C for 30 min and subsequently centrifuged. The absorbance of the supernatant was determined at 532 nm and rectified by subtracting the non-specific absorbance measured at 600 nm. MDA content was measured using the extinction coefficient of 155 mM^–1^cm^−1^ and expressed as nmol g^−1^ fresh weight (Heath and Packer, 1968).

### 2.7 Antioxidant enzymes

Leaf tissue (500 mg) was fused with 0.1 M sodium phosphate buffer solution with PVP for enzyme extraction. Then, the mixture was centrifuged at 14,000*g* for 30 min, and the supernatant was used for various enzymatic component analyses.

#### 2.7.1 Superoxide dismutase assay

The activity of superoxide dismutase (SOD) was analyzed using the method described by Beyer and Fridovich (1987). The enzyme (0.4 mL) was mixed with methionine (20 mM), EDTA (0.15 mM), nitro blue tetrazolium (NBT; 0.12 mM), riboflavin (13 μM), and sodium carbonate (0.05 M). The test tubes containing the mixture were kept under fluorescent lamps for 30 min; the control tubes were not kept under the lamps and served as blank. NBT photoreduction was measured at 560 nm. SOD activity was expressed as the enzyme content required for a 50% decrease in NBT reduction.

#### 2.7.2 Catalase assay

Catalase (CAT) activity was measured by H_2_O_2_ breakdown at 240 nm for 1 min using a 39.4 mM^−1^ cm^−1^ extinction coefficient (Cakmak and Marschner, 1992). The sample contained potassium phosphate buffer (50 mM), EDTA (1 mM), H_2_O_2_ (10 mM), and the enzyme extract (0.2 mL). Enzyme activity was expressed as 1 nmol H_2_O_2_ dissociated min^−1^.

#### 2.7.3 Ascorbate peroxidase assay

Ascorbate peroxidase (APX) activity was assessed using the procedure described by Nakano and Asada (1981). The potassium phosphate buffer (25 mM), EDTA (0.1 mM), ascorbate (0.25 mM), enzyme extract (0.2 mL), and H_2_O_2_ (1 mM) were mixed, and the optical density was measured for 1 min at 290 nm with a 2.8 mM^–^^1^ cm^−1^ extinction coefficient.

#### 2.7.4 Guaiacol peroxidase assay

Guaiacol peroxidase (GPX) activity was estimated using the procedure described by Hemeda and Klein (1990). The solution containing phosphate buffer (25 mM), EDTA (0.1 mM), 0.05% guaiacol, H_2_O_2_ (1 mM), and the enzyme extract was assayed. The absorbance was measured at 470 nm with an extinction coefficient of 26.6 mM^−1^∙cm^−1^.

### 2.8 Statistical analysis

The effects of the treatment were evaluated with a randomized block design with three replications. Statistical significance was tested via an analysis of variance (ANOVA) using SPSS, and the standard error of the mean was analyzed by applying Duncan’s multiple range test (DMRT) at *P* < 0.05.

## 3 Results

### 3.1 Growth variables

Pea seedlings treated with CA had reduced (*P* < 0.05) length and biomass compared with control. Seedling biomass decreased with both CA concentrations, but the effect was higher for the higher CA concentration, CA2. CA2 reduced root and shoot length and fresh and dry biomass by 31%, 12%, 37%, and 51%, respectively (Table 1). SPD treatments partially mitigated the deleterious impact of CA on growth parameters, partially recovering seedling length, and biomass.

**Table 1:** Effect of cinnamic acid (CA) on the germination and growth of *Pisum sativum* seedlings grown with or without spermidine (SPD).

Treatment	Germination (%)	Root length (cm)	Shoot length (cm)	Fresh weight (g)	Dry weight (g)	Relative water content (RWC, %)
Control	98.5 ± 1.27^a^	10.58 ± 0.57^a^	13.21 ± 0.55^a^	3.91 ± 0.29^a^	0.65 ± 0.13^b^	94.32 ± 0.73^a^
SPD	100.0 ± 0.71^a^	12.63 ± 0.79^a^	15.08 ± 0.44^a^	4.28 ± 0.59^a^	0.96 ± 0.10^a^	97.24 ± 0.68^a^
CA1	71.33 ± 0.82^c^	8.95 ± 0.97^b^	12.42 ± 0.59^b^	3.15 ± 0.65^b^	0.48 ± 0.09^c^	85.41 ± 0.49^b^
SPD + CA1	82.5 ± 1.14^b^	9.52 ± 0.78^a^	13.15 ± 0.51^a^	3.98 ± 0.47^a^	0.51 ± 0.18^b^	89.30 ± 0.25^b^
CA2	57.5 ± 0.31^d^	7.27 ± 0.38^c^	11.56 ± 0.74^c^	2.46 ± 0.24^c^	0.32 ± 0.04^d^	78.26 ± 1.36^c^
SPD + CA2	69.0 ± 0.71^c^	8.65 ± 0.99^b^	12.19 ± 0.42^b^	3.21 ± 0.49^b^	0.45 ± 0.01^c^	83.68 ± 0.42^b^

Data are expressed as mean ± standard error of three independent experiments with three replicates. The values followed by different letters indicate a significant difference between treatments according to ANOVA and DMRT.

### 3.2 Pigment content

Pigments are fundamental components for plant maturation and development that regulate metabolic processes. CA had adverse effects on pigment accumulation; CA2 reduced total chlorophyll and carotenoid contents by 38% and 44%, respectively, compared with the control. Foliar application of SPD significantly increased total chlorophyll content by 14% and 21% and carotenoid by 17% and 30% in SPD + CA1 and SPD + CA2, respectively, compared with CA1 and CA2 alone (Table 2).

**Table 2:** CA effect on the pigment content of *Pisum sativum* seedlings grown with or without SPD.

Treatment	Chlorophyll *a* (mg g^−1^ FW)	Chlorophyll *b* (mg g^−1^ FW)	Total chlorophyll (mg g^−1^ FW)	Carotenoids (mg g^−1^ FW)
Control	3.24 ± 0.18^a^	1.26 ± 0.04^a^	4.5 ± 0.45^a^	0.95 ± 0.04^a^
SPD	3.51 ± 0.29^a^	1.42 ± 0.19^a^	4.93 ± 0.47^a^	0.98 ± 0.01^a^
CA1	2.62 ± 0.17^b^	0.95 ± 0.04^c^	3.57 ± 0.36^b^	0.72 ± 0.09^c^
SPD + CA1	2.95 ± 0.16^b^	1.13 ± 0.04^b^	4.08 ± 0.34^a^	0.84 ± 0.13^b^
CA2	1.89 ± 0.08^c^	0.88 ± 0.09^c^	2.77 ± 0.18^c^	0.53 ± 0.07^d^
SPD + CA2	2.34 ± 0.27^b^	1.02 ± 0.04^b^	3.36 ± 0.48^b^	0.69 ± 0.09^c^

Data are expressed as mean ± standard error of three independent experiments with three replicates. The values followed by different letters show a significant difference between treatments according to ANOVA and DMRT.

### 3.3 Biochemical components

CA treatments significantly changed plant biochemical components. Sugar content decreased by 22% and 32% in pea seedlings treated with CA1 and CA2, respectively, compared with the control. SPD + CA1 and SPD + CA2 recovered sugar content values by 9% and 19%, respectively, compared with CA1 and CA2 alone. Protein content decreased in pea seedlings exposed to CA. The effect was concentration-dependent, with CA1 and CA2 treatments reducing protein content by 9% and 23%, respectively, compared with the control. SPD + CA1 and SPD + CA2 treatments recovered CA-only protein values by 4% and 13%, respectively (Table 3). CA also decreased nitrate reductase activity in a dose-dependent manner; the reduction reached 13% and 21% in CA1 and CA2, respectively, compared with the control. Interestingly, SPD stimulated nitrate reductase activity by 10% relative to the control. In combined treatments, the activity significantly increased by 9% and 13% for SPD + CA1 and SPD + CA2, respectively, compared with CA1 and CA2 alone.

**Table 3:** CA effect on sugar, protein, and proline content and the proline content of *Pisum sativum* seedlings grown with or without SPD.

Treatment	Sugar (mg g^−1^ DW)	Protein (mg g^−1^ FW)	Nitrate reductase activity (µmol NO_2_ g^−1^ FW h^−1^)	Proline (µg g^−1^ DW)
Control	3.62 ± 0.12^b^	21.24 ± 0.76^a^	22.69 ± 0.54^a^	20.51 ± 0.63^c^
SPD	4.19 ± 0.48^a^	23.14 ± 0.67^a^	24.86 ± 0.93^a^	18.54 ± 0.54^d^
CA1	2.81 ± 0.18^c^	19.41 ± 0.46^b^	19.68 ± 0.98^c^	25.85 ± 1.31^b^
SPD + CA1	3.05 ± 0.15^b^	20.19 ± 0.83^b^	21.36 ± 0.96^b^	26.33 ± 0.41^b^
CA2	2.46 ± 0.22^c^	16.34 ± 0.78^c^	17.94 ± 0.99^d^	28.92 ± 0.92^a^
SPD + CA2	2.93 ± 0.54^c^	18.54 ± 0.52^b^	20.22 ± 0.57^b^	29.13 ± 0.18^a^

Data are expressed as mean ± standard error of three independent experiments with three replicates. The values followed by different letters indicate a significant difference between treatments according to ANOVA and DMRT.

Proline content increased in pea leaves treated with CA compared with the control. SPD further increased proline by 2% and 1% in SPD + CA1 and SPD + CA2 treatments, respectively.

### 3.4 Oxidative stress variables

**Fig. 1:**
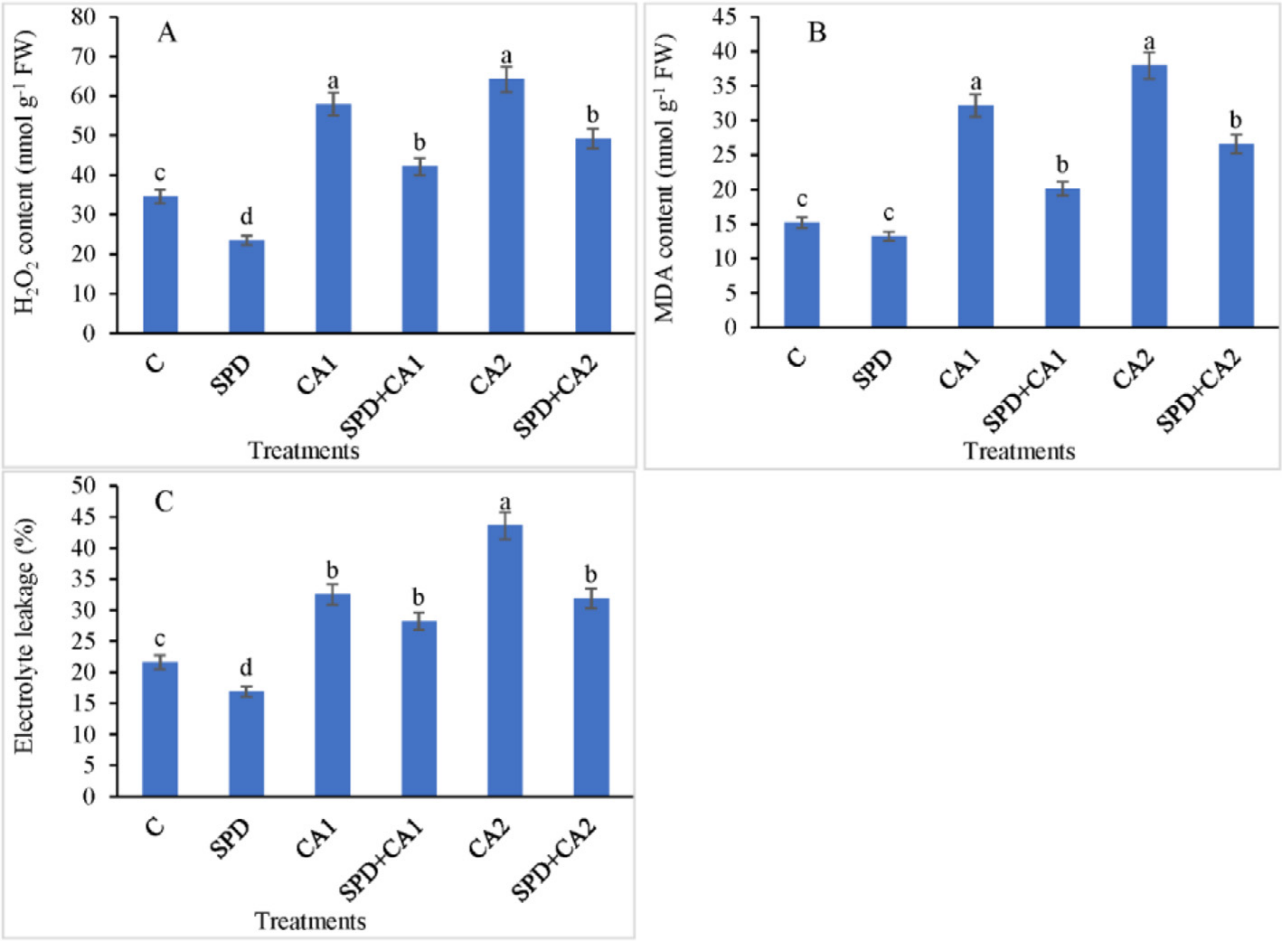
Effect of cinnamic acid (CA) on (A) hydrogen peroxide (H_2_O_2_) content, (B) MDA content, and (C) electrolyte leakage in *Pisum sativum* L. grown with or without spermidine (SPD). Data are expressed as mean ± standard error of three independent experiments with three replicates. The values followed by different letters indicate a significant difference between treatments according to ANOVA and Duncan’s multiple range tests. SPD = 1.0 mM, CA1 = 1 mM, and CA2 = 1.5 mM.

CA1 and CA2 treatments increased H_2_O_2_ content by up to 67% and 85% compared with the control (Fig. 1A). The MDA content also gradually increased in leaves treated with CA. The results indicate that SPD exposure reduced the MDA content in CA1 and CA2 treatments by 37% and 30%, respectively, emphasizing the shielding function of SPD (Fig. 1B). Electrolyte leakage was higher with CA1 and CA2 treatments, increasing by up to 50% and 102%, respectively, compared with the control. SPD application in CA1 and CA2 treatments reduced electrolyte leakage by 13% and 27%, respectively (Fig. 1C).

### 3.5 Antioxidant enzymes

**Fig. 2:**
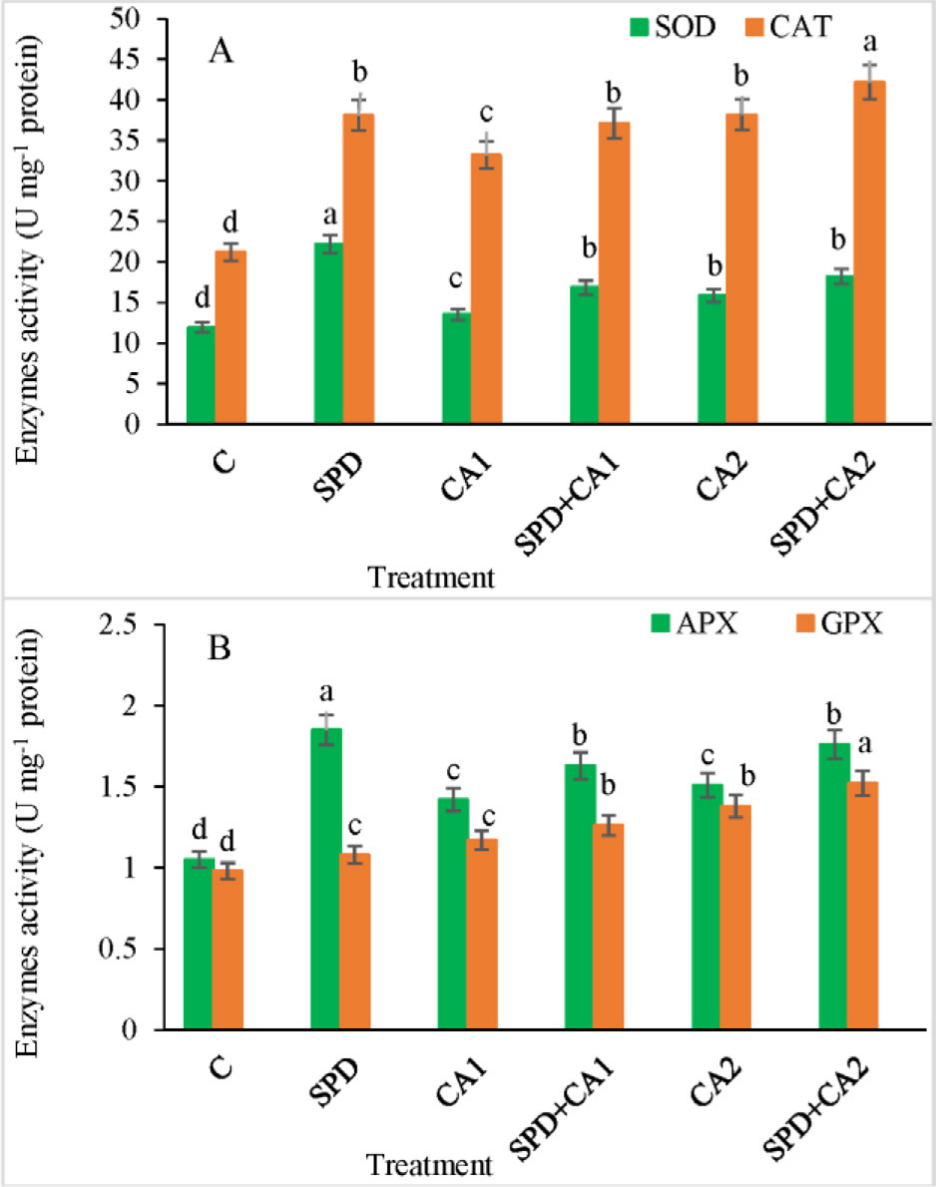
Impact of CA on the activity of antioxidant enzymes (A) SOD and CAT and (B) APX and GPX in *Pisum sativum* L. grown with or without SPD. Data are expressed as mean ± standard error of three independent experiments with three replicates. The values followed by different letters indicate a significant difference at P < 0.05 between treatments according to the ANOVA and DMRT. SPD = 1.0 mM, CA1 = 1 mM, and CA2 = 1.5 mM.

Treatment of pea leaves with CA alone or in combination with SPD significantly affected the antioxidant system. The antioxidant enzymes responded differently when the seedlings were treated with CA or with CA + SPD (Fig. 2A–B). SOD, CAT, APX, and GPX activities increased in seedlings exposed to graded CA concentrations (CA1 and CA2). Interestingly, SPD + CA treatments led to higher SOD and CAT activities than CA alone (Fig. 2A). The application of SPD with CA also increased APX and GPX activities more than CA treatments. SPD + CA1 and SPD + CA2 treatments led to significant increase in SOD, CAT, APX, and GPX activities by 25%, 12%, 15%, and 8% and 15%, 11%, 17%, and 10% relative to CA1 and CA2, respectively.

## 4 Discussion

Plants are exposed to different unfavorable environmental conditions. CA, a phenolic acid, acts as an allelochemical that reduces germination and impairs plant development, affecting various physiological pathways (Salvador et al., 2013). Our results indicate the hormetic effect of phenolic compounds on plant growth and metabolism (Hussain et al., 2017). Gelsomino et al. (2015) reported that the application of CA reduced root growth in maize plants. Therefore, various approaches have been developed to minimize the adverse impacts of CA by reducing its absorption or boosting the mechanisms of plant tolerance. Polyamines have been used as additives against the detrimental effects of phenolic compounds on crop plants. Here, we reported the beneficial effects of SPD in ameliorating the damaging effects of CA on *Pisum sativum* seedlings*.*

CA treatments reduced seedling length, biomass, and pigment content relative to the control, with the strongest effects obtained with CA2. CA also reduced protein content and nitrate reductase activity. Indoleacetic acid has crucial functions in seedling growth and modulates root and shoot architecture, cellular organelle patterning, and vascular system development. CA inhibits the growth-enhancing effect of indoleacetic acid and increases the rate of IAA degradation by promoting IAA oxidase activity (Einhellig, 2004). CA negatively affects plant growth by disrupting the cell membrane, changing xylem and phloem anatomy, increasing lipid peroxidation, inducing oxidative stress-related free radical production, inhibiting nitrate uptake, and reducing plasma membrane H^+^-ATPase activity (Abenavoli et al., 2010). These changes can then disrupt photosynthesis, respiration, sugar and protein metabolism, and enzyme activities (Weir et al., 2004, Hussain et al., 2017).

The cell wall is the plant’s first line of defense and is thus susceptible to allelochemical-mediated stress. Allelochemicals reduce root growth by inducing lignin deposition in the cell wall, which leads to structural modifications in the root cells (Hussain et al., 2017). CA treatments inhibited pea seedling growth by interfering with metabolic activities and consequently reducing cell division and elongation (Einhellig, 2004, Salvador et al., 2013). In addition, CA disrupted plant–water relations and reduced the RWC in leaves (Table 1). This effect was stronger for the higher dose, CA2. SPD partially recovered the RWC of CA-treated plants by promoting osmotic acclimatization or changing the stretchiness of the cell wall (Dashtmian et al., 2014). The accretion of osmolytes facilitates the maintenance of cell water potential and promotes water uptake by lowering the water potential (Ahanger et al., 2020). Combined treatment (SPD + CA) was applied to determine whether SPD alleviated the negative impacts of CA-generated allelopathic stress. Plant exposure to growth promoters causes cell wall extension and results in cell elongation and expansion, thus enhancing seedling growth. Hence, SPD likely counteracts the inhibitory effects of CA by promoting pea seedling growth (Roychoudhury et al., 2011).

The CA-induced decline in pigment content might be due to the reduction of ribulose 1,5-bisphosphate carboxylase/oxygenase efficiency, changes in chloroplast structure, and degradation of proteins in the membrane of the photosynthetic apparatus (Gengmao et al., 2015). The exogenous application of SPD reduces chlorophyll degradation by decreasing chlorophyllase activity, stabilizing the chloroplast structure (Li et al., 2015, Yadav et al., 2018). CA reduced the photosynthetic rate and RuBPC activity in cowpea (Huang and Bie, 2010). In addition, it reduced photosystem II efficiency in *Lactuca sativa* leaves (Hussain and Reigosa, 2011). CA-induced allelochemical stress interfered with the synthesis of porphyrin, a chlorophyll *a* precursor, and resulted in the reduced accumulation of photoassimilates, leading to reduced pea seedling growth (Table 1).

Protein synthesis is associated with the production of new tissues and is the main source of nitrogen compounds. The lower protein content in leaves treated with CA was caused by reduced protein biosynthesis and enhanced degradation (Hussain et al., 2017). Nitrogen is an integral part of chlorophyll and proteins. It is absorbed by plants as nitrate, which helps in plant development. Nitrate reductases reduce nitrate into nitrite. CA significantly decreased nitrate reductase activity in pea leaves. Such a decrease was associated with the changes in protein synthesis. Nitrate absorption is suppressed by phenolic acids, with adverse effects on plant growth. Such a reduction could also result from reduced carbon fixation, root nitrate absorption, or nitrate availability to seedlings (Kleinhofs and Warner, 1990). In this study, CA inhibited nitrate reductase activity, but the addition of SPD enhanced the contents of total protein and nitrate reductase in leaves compared with CA1 and CA2 alone (Table 3). Osmolytes function as signaling molecules that mitigate the adverse impact of stress on physiological cycles by preserving enzyme activity and the photosynthetic oxygen-evolving complex and maintaining the oxidation–reduction balance (Ahanger and Agarwal, 2017). Plants with high osmolyte content have better stress response capacity and free radical-removing abilities (Ahanger et al., 2019). SPD significantly enhanced sugar contents in CA stressed seedlings by promoting the removal of free radicals and inhibiting photoinhibition (Cuin and Shabala, 2007). Proline, a compatible solute, acts as an antioxidant, removing ROS, and preserving cellular machinery. In our results, the increase in proline levels in SPD + CA1 and SPD + CA2 treatments reflects the stress-alleviating role of SPD (Roychoudhury et al., 2011). Tanou et al. (2014) revealed that high proline content achieved by polyamine application results from the changes in its gene expression.

Cell membrane disruption and lipid degradation are major stress indicators, which change plasma membrane impaling capacity and fluidity (Zhang et al., 2010). The increase in electrolyte leakage observed in CA-treated seedlings indicates cell membrane damage (Hussain et al., 2017). The MDA content is a marker of cell membrane lipid peroxidation. Hence, the CA-dependent increase in the MDA content indicates the presence of oxidative stress derived from the overproduction of ROS (Ding et al., 2007); SPD supplementation reduced lipid peroxidation. CA also increased H_2_O_2_ production, which enhanced lipid peroxidation (Ding et al. 2007). However, SPD diminished H_2_O_2_-induced lipid peroxidation by lowering H_2_O_2_ concentration (Ahanger et al., 2018).

Plant responses to different environmental constraints elevate oxidative stress by generating excessive free radicals, which damage biomolecules, ultimately causing cell death. Antioxidant enzymes play a significant role in plant protection and can alleviate free radicals, preventing lipid peroxidation and maintaining cell membrane structure (Fimognari et al., 2020). CA1 and CA2 treatments increased the activity of the antioxidant enzymes SOD, CAT, APX, and GPX. The enhanced antioxidant activity in pea leaves resulted from the increase in free radical production and oxidative impairment. SPD played an important defense role by suppressing the oxidative stress consequences induced by CA (Fig. 2A–B). SOD participates in a frontline protective mechanism that converts free radicals to H_2_O_2_, whereas CAT eliminates H_2_O_2_ by changing it to H_2_O. GPX regulates the conversion of H_2_O_2_ into H_2_O and also modulates cell wall properties during plant expansion. Antioxidant enzyme activity increased in *Solanum lycopersicum* exposed to phenolic compounds (Hussain et al., 2017), which is in agreement with our results in pea.

## 5 Conclusion

In this study, SPD alleviated the adverse effects of CA stress on pea proliferation and biochemical parameters by increasing antioxidant concentration and reducing the level of free radicals and the consequent oxidative damage to biomolecules. The application of SPD increased the pigment and protein contents and nitrate reductase activity in seedlings by promoting antioxidant enzyme activity. Hence, SPD acts as a regulator of redox homeostasis and functions in oxidative stress management in pea seedlings. Future research should investigate the molecular mechanisms involved in SPD-triggered plant stress resistance.

## Ethics approval

Not Applicable.

## Consent to participate

All authors consent to participate in this manuscript.

## Consent for publication

All authors consent to publish this manuscript in Saudi journal of Biological Science.

## Availability of data and material

Data will be available on request to corresponding or first author.

## Code availability

Not Applicable.

## Author contribution

RTK conceived the idea and performed the experiments. MNA and PA analyzed the data and revised the manuscript

## Declaration of Competing Interest

The authors declare that they have no known competing financial interests or personal relationships that could have appeared to influence the work reported in this paper.
